# Integrated care as a means to improve primary care delivery for adults and adolescents in the developing world: a critical analysis of Integrated Management of Adolescent and Adult Illness (IMAI)

**DOI:** 10.1186/1741-7015-12-6

**Published:** 2014-01-14

**Authors:** Ashwin Vasan, Andrew Ellner, Stephen D Lawn, Sandy Gove, Manzi Anatole, Neil Gupta, Peter Drobac, Tom Nicholson, Kwonjune Seung, David C Mabey, Paul E Farmer

**Affiliations:** 1 Program in Global Primary Care and Social Change, Department of Global Health & Social Medicine, Harvard Medical School, Boston, MA, USA; 2 Partners In Health, Boston, MA, USA; 3 Partners In Health, Kigali, Rwanda; 4 Department of Clinical Research, Faculty of Infectious and Tropical Diseases, London School of Hygiene & Tropical Medicine, London, UK; 5 Department of Medicine, Weill Cornell Medical College/New York-Presbyterian Hospital, New York, NY, USA; 6 Division of Global Health Equity, Brigham & Women’s Hospital, Boston, MA, USA; 7 IMAI-IMCI Alliance, San Francisco, CA, USA

**Keywords:** Primary care, Integrated management, Integration, Quality improvement, Health care delivery, Health systems, IMAI

## Abstract

**Background:**

More than three decades after the 1978 Declaration of Alma-Ata enshrined the goal of ‘health for all’, high-quality primary care services remain undelivered to the great majority of the world’s poor. This failure to effectively reach the most vulnerable populations has been, in part, a failure to develop and implement appropriate and effective primary care delivery models. This paper examines a root cause of these failures, namely that the inability to achieve clear and practical consensus around the scope and aims of primary care may be contributing to ongoing operational inertia. The present work also examines integrated models of care as a strategy to move beyond conceptual dissonance in primary care and toward implementation. Finally, this paper examines the strengths and weaknesses of a particular model, the World Health Organization’s Integrated Management of Adolescent and Adult Illness (IMAI), and its potential as a guidepost toward improving the quality of primary care delivery in poor settings.

**Discussion:**

Integration and integrated care may be an important approach in establishing a new paradigm of primary care delivery, though overall, current evidence is mixed. However, a number of successful specific examples illustrate the potential for clinical and service integration to positively impact patient care in primary care settings. One example deserving of further examination is the IMAI, developed by the World Health Organization as an operational model that integrates discrete vertical interventions into a comprehensive delivery system encompassing triage and screening, basic acute and chronic disease care, basic prevention and treatment services, and follow-up and referral guidelines. IMAI is an integrated model delivered at a single point-of-care using a standard approach to each patient based on the universal patient history and physical examination. The evidence base on IMAI is currently weak, but whether or not IMAI itself ultimately proves useful in advancing primary care delivery, it is these principles that should serve as the basis for developing a standard of integrated primary care delivery for adults and adolescents that can serve as the foundation for ongoing quality improvement.

**Summary:**

As integrated primary care is the standard of care in the developed world, so too must we move toward implementing integrated models of primary care delivery in poorer settings. Models such as IMAI are an important first step in this evolution. A robust and sustained commitment to innovation, research and quality improvement will be required if integrated primary care delivery is to become a reality in developing world.

## Background

The 1978 Declaration of Alma-Ata [1], where member states of the World Health Organization (WHO) declared ‘health for all’ by the year 2000, remains a significant, if elusive, goal in global health. Spurred by limited successes in community-based primary care delivery in India, China, and elsewhere, health ministers and policymakers from 134 countries gathered at the International Conference on Primary Healthcare at Alma-Ata (now Almaty, Kazakhstan) to express their commitment to bringing primary care services to scale around the globe [2]. That vision of comprehensive global primary care, while still unrealized, informs our current concept of primary care as a system that encompasses the provision of basic medical services and also accounts for community needs [3]. Alma-Ata also explicitly acknowledged the often overlooked links between primary care and broader issues of social protection such as clean water and sanitation, nutrition, and food security [4]. As we continue to combat global health inequalities, we can learn from Alma-Ata and from the subsequent implementation (or lack thereof) of primary care programs in resource-poor countries [5-8].

The decades since Alma-Ata have witnessed dramatic shifts in priorities, political will, and funding in global health. Amidst such shifts, there have been limited successes, including encouraging improvements in child survival and immunization coverage for vaccine-preventable diseases [9]. The Millennium Development Goals (MDGs), particularly the health-related goals on child survival, maternal health, HIV, tuberculosis, and malaria, laid out benchmarks for primary health systems performance. The proliferation of global health initiatives for vertical, disease-specific programs could, and should, be leveraged for wider systems improvement [10,11]. Despite Alma-Ata’s clarion call, primary care continues to attract too few resources. The health-related Millennium Development Goals remain out of reach for many countries in the developing world, particularly in sub-Saharan Africa where shortages of doctors, nurses, and community health workers threaten the advances that have been made [12,13]. This paper first attempts to outline several sources of the persistent dissonance between primary care theory and practice as they apply to effective global primary care delivery. Secondly, this paper examines the role of service integration and integrated models of care and their potential to revitalize and strengthen primary care delivery in poor settings. Finally, the paper examines the WHO’s Integrated Management of Adolescent and Adult Illness (IMAI) as one example of such a model that is deserving of consideration as a method to improve basic primary care delivery.

## Discussion

### Alma-Ata and the politics of language

Despite advancing the primary care discourse as a whole, Alma-Ata lacked a clear implementation plan and failed to bring about operational consensus, instead focusing entirely on ‘primary healthcare’ as a high concept encompassing intersectoral approaches with a distinct community and sociopolitical focus [1]. In this vacuum, artificial distinctions emerged between ‘primary healthcare’, as above, and ‘primary care’ as simply front-line clinical services [2,14-16]. Some have subsequently tried to bring clarity to primary care’s role as both a discipline and a practice. Frenk, for example, differentiates three uses of the word ‘primary’ [17] as (1) first contact: the point of first contact between the patient and the formal health system, (2) first level: the preventive and curative services delivered at the front line of a health system, and (3) first causes: the social determinants of health and the interdisciplinary approaches to addressing basic public health needs [18]. Starfield and Shi suggest that the distinction between primary care as a service within the broader schema of primary healthcare is critical in order to mobilize societal actions towards health equity [19] and Gilson *et al*. put forward that good primary care delivery is dependent on robust primary healthcare [20]. More recently, others have tried to cast primary care as a distinct medical specialty, a set of functions within the health system, or a way to orient health systems through regional-level or area-level aggregates [21]. It would appear that none of these distinctions have achieved broad consensus.

As academic circles debated the theoretical definitions of primary care, funders, policymakers, and implementers shifted away from Alma-Ata’s broad and systems-oriented vision in favor of programs more limited in scope and thus deemed more feasible, rapid, and measurable [22]. This is, in part, the reason that ‘primary care’ often refers to targeted initiatives (immunization campaigns, for example) as opposed to more complex systems interventions.

### Integration and gaps in adult and adolescent primary care

In recent years, calls have been made to capitalize on the successes of vertical interventions by increasing investment in broader global health targets [23], particularly better integration. Integration, like primary care itself, has been defined from multiple perspectives: from the perspective of the multiple government sectors and academic disciplines that impact primary care, from the perspective of the health system, and from the perspective of disease, meaning the combination of clinical services for specific diseases or groups of diseases into integrated, essential packages of care. However, from any perspective, the challenge remains in delivering front-line primary care services that optimize both coverage and equity without compromising quality of care.

The literature suggests that concise and integrated clinical management guidelines can play a role in improving the quality of comprehensive primary care delivery [24], much as standardized guidelines and protocols have improved quality within individual global health initiatives [11]. In many instances, this type of integration has involved integrating HIV/AIDS services into other existing programs. For instance, integration of HIV care with tuberculosis (TB) and sexually transmitted infection (STI) services in Haiti showed demonstrable increases in patient access, uptake of testing and case detection, enrollment in antiretroviral therapy (ART), and even benefits in unrelated programs such as vaccine coverage [25-27]. Similar results with TB/HIV integration have demonstrated improved rates of screening [28,29] increased enrollment in antiretroviral treatment programs [30], and even improved TB treatment outcomes in one cohort in Ghana [31], but a recent systematic review of TB/HIV integration suggested that more robust downstream outcome measures should be emphasized in future research [32]. Research has also been conducted on integration of HIV care and treatment services with programs such as Prevention of Mother to Child Transmission (PMTCT) [33] and family planning services [34-36], with generally positive results with respect to patient access, and patient and provider perceptions. But not only are these outcomes limited in scope and generalizability, these integrated programs also benefit from the fact that HIV is coincident or closely related with these conditions/services and as such binary integration of two or several related programs may not be applicable to the wider primary care setting. There are several studies examining integrated HIV care with routine primary care services [25,37,38], but again most examine access, uptake, and systems metrics without reporting impact on clinical care and treatment for other general primary care conditions, or overall patient outcomes. Finally, there have been numerous examples of binary program integration between non-HIV disease programs and routine primary care, previously and comprehensively reviewed [39-41], but many do not report quantitative outcomes or outcomes that enable robust comparisons and conclusions about integration as a quality improvement strategy.

A more general approach to integration has been implemented and studied, however, with some successful examples. Based on an early version of IMAI and initially designed as an intervention to integrate acute respiratory conditions and HIV care into routine primary care services in South Africa [42,43], the Practical Approach to Lung Health in South Africa (PALSA) was expanded to include a range of common primary care conditions [44] and rigorously tested and scaled up nationally to over 10,000 nurse-led primary care teams (M Zwarenstein, Institute for Clinical Evaluative Sciences, University of Toronto personal communication) and internationally in Malawi [45,46] where ongoing testing continues. Results from implementation of this program has shown not only gains in specific outcomes such as rates of cotrimoxazole prophylaxis for PCP and increase rates of TB diagnosis in the primary care setting [47], but also improved provider perception of care [44,48] and even cost effectiveness [49].

Another notable example of integrated clinical management at the point of delivery is the WHO/United Nations International Children’s Emergency Fund (UNICEF) Integrated Management of Childhood Illness (IMCI) for children under 5. The IMCI framework has proven successful in integrating treatment for specific diseases (for example, acute respiratory infections, anemia/malnutrition, diarrheal disease, malaria) and preventive interventions into a single integrated guideline with the aim to address the major causes of childhood mortality in children under 5, when delivered by well-trained and well-supported multipurpose health workers. In addition to improving outcomes for specific conditions such as pneumonia [50], the implementation of IMCI has also led to reductions in overall childhood mortality and overall cost savings to the health system [51,52]. Although the multi-country evaluation of IMCI raised concern about health system limitations to expanding IMCI implementation and the lack (then) of community tools [53] and while there has been debate around issues of inadequate ongoing support and missed opportunities for quality improvement [54], newer long-term data from Egypt also suggests that under-5 care based on IMCI leads to durable mortality reductions [55], and more than 100 countries continue to implement IMCI as a key component of primary care delivery to children under 5 years of age.

In general, the impact of integration on primary care delivery is poorly understood, particularly for adults and adolescents. While the data above suggest that integrated clinical and program management for specific (and often related) conditions could be an important strategy in improving quality of primary care delivery, the overall quality of data is generally poor and, often based on incomparable outcomes. Definitive reviews have explicitly called for expanded and improved research in the field [39-41]. In addition, little study has occurred on how to implement a standard and integrated approach to the initial general patient assessment and screening in primary care clinics in poor settings. Such a standard approach could serve as the basis for the delivery and ongoing quality improvement of integrated primary care for populations in these settings.

### The state of adult and adolescent primary care delivery

Particularly in low-income nations with tiered health systems, the majority of adult primary care is delivered in outpatient departments (OPDs), a legacy often attributed to structural recommendations initially put forth in the UK Dawson Report of 1920 [17] (see Box 1). In many resource-limited countries, particularly in rural areas, these departments serve as catchalls, delivering general and limited specialty care together while focusing on management of acute conditions. The care provided to adults and adolescents in outpatient departments, however, often lacks the necessary standardization and integration, apart from disease-specific services delivered within OPDs. Beyond protocols in national guidelines, there is little reference material available for use during the general patient consultation and when available, these materials are usually disease specific. Charting is often performed in a single-lined register, which reflects a troubling pattern of minimal data collection, and only acute, episodic, and a very limited scope of care. Little is known of whether nurses appropriately screen for sexually transmitted infections or for tobacco or alcohol abuse, for instance, or provide appropriate counseling on prevention and lifestyle changes. So, as expected, there has been little rigorous study on the quality of adult and adolescent primary care delivery in the developing world from either the clinical or operational perspective, with most research focusing instead on self-reported or self-perceived performance [56], or using metrics from children under 5 as a proxy for overall effectiveness of primary care within the health system [57]. Perhaps most importantly, there is often no mention of attempts at iterative changes or improvements. It is ironic that this paucity of data limits the resources devoted to further examination, perpetuating major gaps in understanding in this important area of healthcare delivery in poor settings.

### Box 1: Functions of the OPD in the health system*

• Triage and referral or admission of acutely ill patients.

• Diagnosis and management of acute, non-severe illness that does not require hospitalization or higher-level care (for example, diarrhea, pneumonia, malaria).

• Screening, diagnosis, and management or referral of chronic illnesses (for example, HIV or non-communicable chronic diseases) that may require longitudinal specialty care from a clinic or service that deals with these conditions specifically.

• Provision of health behavior messages and prevention services, including for family planning, STI and HIV prevention, nutritional counseling and support, as well as lifestyle modification for chronic disease prevention.

*Some variation by particular setting or country.

### IMAI as an example of integrated primary care for adults and adolescents

We are neither the first to recognize the need for standardization and integration in adult primary care delivery, nor the first to propose the development and implementation of essential packages of care [9]. Capitalizing on the success of IMCI, WHO developed IMAI in a similar fashion, first as a series of simplified, syndromic case management protocols to diagnose and manage common adult illnesses in resource-poor settings, and then integrated into a single clinical management guideline [58]. The IMAI acute care protocols are structured around presenting symptoms, and classify the patient according to clinical severity and disease chronicity using a syndromic approach structured around a simplified version of the universal patient history and physical examination. This is followed by simple and prescriptive algorithms for syndromic treatment as well as follow-up and/or referral recommendations. Like IMCI before it, IMAI draws upon proven approaches to the screening, diagnosis, and management of specific diseases including malaria, HIV/AIDS, STIs, pneumonia, diarrheal disease, and tuberculosis. IMAI was introduced to improve acute care through better integration of delivery at a single point-of-care, usually health center or hospital OPDs. It was designed to target nurses and other providers delivering care at the front lines of health systems. It was the one of the first guidelines of its kind to address adult primary care in both general and integrated fashions, and to take proven interventions for priority diseases and present them within a unified strategy.

With unprecedented attention and funding for HIV/AIDS at the time of IMAI’s initial development, IMAI’s implementation was absorbed within the broader movement to increase access to antiretroviral therapy. WHO developed the IMAI *Chronic HIV Care with ART* handbook [59] to build health worker capacity in managing HIV disease with antiretroviral therapy (ART), using a simplified format analogous to the original IMAI protocols, and this handbook quickly became the most visible and widely implemented component of the IMAI package, particularly in southern and eastern Africa. It did not offer new management protocols *per se*, but rather incorporated existing WHO clinical staging, chronic HIV care, antiretroviral therapy and prevention guidelines into a simpler format targeting nurses and multipurpose healthcare workers operating within decentralized ART scale-up programs [60,61],. It was based on applying the general principles of good chronic care [ref], derived from review of the experience with non-communicable disease programs, and was innovative in its use of PLHIV expert patients, both on the clinical team and as trainers.[ref]. Currently, more than 40 countries are in various stages of adaptation or implementation of IMAI for use in their HIV treatment programs.

Building on the lessons learned in scaling up AIDS treatment and care, the original IMAI *Acute Care* guidelines continued to evolve and expand, yet have not been implemented with nearly the same support. Consequently, the majority of available research on IMAI to date has focused on the *Chronic HIV Care* guidelines, which does not offer insight into IMAI’s potential to improve primary care delivery through integration [62,63]. The limited available literature on the original *Acute Care* components of IMAI has shown mixed but generally promising results, though they remain mostly unpublished. For example, an unpublished 2003 validation study using the cough and/or difficulty breathing algorithm showed a sensitivity of 72% in detecting severe pneumonia, but was insensitive (0% to 22%) in detecting other causes of respiratory illness, and non-specific in its detection of chronic pulmonary conditions [Simoes E, Todd J, English R, Sepulveda R, Ottomani SE, Gove S: Preliminary Analysis of IMAI Validation Studies. 2003. Unpublished.]. A 2009 multicenter study utilizing the acute care algorithms in an HIV-positive cohort at Ethiopian government health centers demonstrated greater than 85% sensitivity and greater than 92% specificity in diagnosing upper respiratory tract infection, pneumonia, tuberculosis, and dysentery [64]. The algorithm performed poorly, however, in assessing the severity of illness and in the diagnosis and assessment of anemia. Finally, a recent study from Lesotho showed that a number of specific symptoms and clinical signs from the algorithm were significant predictors of different disease states (for example, chronic vs acute respiratory conditions, tuberculosis, pneumonia), but overall only moderately sensitive and specific [Seung KJ, Rigadon J, Finch M, Gove S, Vasan A, Ramangoaele L, Satti H: Evaluation of integrated management guidelines for patients with respiratory symptoms. 2011. Unpublished]. The only known research on IMAI *Acute Care* to date, these three studies, while certainly revealing some mixed results, indicate the potential for the IMAI algorithms to positively impact general adult acute primary care. Nevertheless, this is certainly a limited evidence base and further investigation is paramount.

### Limitations and opportunities for integrated care and IMAI

There are a number of possible explanations for why IMAI has struggled to achieve programmatic or research relevance in the wider public health and primary care implementation and research agenda. The first hurdle is clinical and technical, reflecting limitations of the basic IMAI syndromic approach. Use of a syndromic management in adults and adolescents is fraught with challenges when compared with children, where it is effective precisely because children frequently present non-specifically and with overlapping clinical signs, where IMCI could focus on a limited number of conditions causing a significant proportion of mortality, and because children are often incapable of providing reliable and detailed histories. Adults, by contrast, usually present with more complex spectra of diseases and etiologies, thus decreasing the utility of broad-spectrum diagnosis and management and increasing the complexity of an integrated clinical algorithm, and limiting the generalizability of a single-integrated guideline across settings with varying epidemiology and demographics, without rigorous adaptation.

The second challenge is that the ‘adult and adolescent’ population is difficult to isolate, both politically and programmatically. It becomes challenging to generate the necessary advocacy and funding for implementation, quality improvement, and research without a clear and defined target population, such as ‘children under 5’ or ‘patients with HIV’. Introduced in the wake of the so-called ‘child survival revolution’ of the 1980s [9], IMCI integrated the major important vertical interventions targeting the under-5 population (nutrition, immunization, acute respiratory infection, malaria, and diarrheal disease programs, for example), thus making it a circumscribed and attractive target for funders, implementers, researchers and policymakers alike and may explain why even today under-5 metrics are often used to describe overall health system performance [65,66]. Moreover, as previously suggested above, the ‘integration’ of closely related or coincident conditions may not be of relevance for a more generalized approach. The numerous and varied vertical programs broadly targeting ‘adults and adolescents’ and impacting primary care delivery, make service integration in this population much more complex, and thus measuring the impact of an intervention such as IMAI on ‘adult survival’ is harder than corresponding interventions for under 5 s, for example.

Third, while IMAI addresses healthcare delivery at the facility level, it does not address the community health and policy interventions necessary for comprehensive primary healthcare as outlined at Alma-Ata. Community health programs and community health workers have been shown to play a critical role in reducing maternal and neonatal morbidity and mortality, as well as in more complex programs such as tuberculosis control and therapy, and ART delivery [67-76]. Additionally, large-scale global efforts are underway to recruit, train, and retain community health workers towards achieving the health-related MDGs [77]. But aside from guidelines on community-based palliative care, patient self-management and ART treatment supporters, the current version of IMAI does not deal explicitly with the integration of facility and community-based primary care nor the training of community health workers to support integrated primary care delivery.

Finally, we must return to the issue of persistent confusion around the aims, definitions, and scope of global primary care. The inability of the public health community to achieve a common operational understanding of primary care, especially for adults and adolescents, has predictably led to inertia in program implementation and difficulty generating a policy consensus, advocacy platform, and funding base. Interventions such as IMAI struggle to find programmatic footing in such a climate [78].

Despite the challenges and potential limitations, we contend that specific models such as IMAI can and should stimulate dialogue regarding the wider use of integrated clinical guidelines to improve primary care delivery in developing countries (see Box 2). Specifically, attention should be given to those models that offer realistic approaches for healthcare workers to provide integrated management for a range of conditions at a single point-of-care. Integrated clinical models such as IMAI take proven clinical approaches for specific illnesses in specific populations and integrate them into a single guideline implemented at a single point-of-care. This type of integration streamlines services for the patient and harmonizes the monitoring, evaluation, and reporting for these conditions. As such, it could be an important catalyst in developing a common standard of global primary care delivery that can serve as the basis for ongoing quality improvement.

IMAI in its current form outlines a preliminary model for integrating acute and chronic care by incorporating screening for HIV, tuberculosis, cardiovascular disease, and chronic respiratory conditions, for example. Long recognized as integral to a comprehensive primary care reform strategy [79], non-communicable disease (NCD) interventions have been integrated into existing vertical delivery for TB [80,81], HIV [82] and reproductive health services [83], but these have been limited in scope and have yet to tackle the more general integration of acute and chronic care into a single approach. Employing integrated and systematic screening for NCDs within a standardized approach to the patient offers a clear and operational entry point into care for patients with chronic diseases who may otherwise be overlooked in a system designed principally for acute care and episodic patient contact. Additionally, the IMAI *General Principles of Good Chronic Care*[84] was developed to draw on the experience of non-communicable disease control to support HIV/AIDS treatment scale up by reorienting existing health worker practices and communication toward longitudinal patient care, but these principles could readily be extended to care for other chronic NCDs going forward. Models such as IMAI could improve follow-up care and referral services by advising providers on when and how to follow an episode of acute illness longitudinally, especially if they are concerned that persistent symptoms may indicate an underlying chronic illness. Finally, IMAI *Acute Care* contains a general prevention section, encompassing such topics as safe sex practices, immunization and the use of bed nets to prevent the transmission of malaria, and thus takes the first practical steps toward integrating prevention and treatment across a range of conditions within a single structured protocol.

### Box 2: Specific ways in which IMAI could advance primary care delivery

1. Integration of services for existing vertical programs

• Integrate proven clinical protocols for specific illnesses such as malaria, STIs, and pneumonia.

• Advance towards healthcare worker in multipurpose settings managing a range of conditions.

• Streamline patient services with potential increase in patient satisfaction and uptake of care.

• Harmonize monitoring, evaluation, and reporting for these conditions.

2. Expanded standardization/protocolization of care

• New, rigorously-developed syndromic protocols for common illnesses such as adult headaches, oral and throat problems, and skin conditions that do not currently have vertical programs.

• Additional chronic care guidelines and training tools for decentralized management of important chronic non-communicable diseases including diabetes, hypertension, and heart failure.

3. Integration of acute and chronic care

• Integrated chronic disease (chronic infections and NCDs) screening within each protocol (for example, HIV, TB, heart disease, chronic lung disease).

• Well-defined entry point into health system for patients with NCDs.

• Guidelines for follow-up of acute illness, moving away from episodic to longitudinal care.

4. Integration of prevention and treatment

• Integrated screening and prevention messages within each protocol, such as tobacco cessation for smokers with chronic cough and respiratory conditions.

• Section on general prophylaxis and behavior modification for routine use, including topics such as condoms and safe sex practices for HIV and STI prevention, tobacco and alcohol cessation, family planning, and use of bednets for malaria prevention.

• Section on prevention in special populations, including adolescents and men who have sex with men (MSM).

### The way forward

There is no doubt that the evidence base for IMAI is weak. And, as previously noted, even the literature on integrated care more generally has significant limitations. But in itself these limitations should not preclude further examination of integrated primary care as a strategy to improve primary care delivery. However flawed, the evidence base for integrated care in under 5 s and in leveraging HIV programs as an entry point to integrated care, at least offers some initial promise for more robust analysis of general primary care approaches. The following specific areas of implementation and research are critical to improving our understanding of the potential of integrated primary care models to improve quality:

1. Describing the baseline: in our review of the peer-reviewed and grey literature we were unable to identify any current data that define quality of care for general adult and adolescent ambulatory acute care at outpatient departments using quantitative approaches. Our group has completed a baseline assessment of OPD care at eight health centers in one district in southeast Rwanda [85], but this must be replicated in multiple settings with varying epidemiology in order to establish quality gaps and to better define the specific role of integrated care in different environments.

2. Implementing the models: robust support must be given for implementing integrated models of care to drive quality improvement for adult ambulatory acute care in primary care clinics. This will require strong didactic training programs, implementation support from Ministries of Health and non-governmental partners working together, and a robust follow-up, supervision, mentoring and quality improvement apparatus to ensure that post-training improvements in quality are sustained. In our project in Rwanda, for example, an integrated mentoring and supervision infrastructure has been implemented to support a range of primary care service delivery at rural health centers in three districts [86].

3. Validating the models: one of the strengths of the syndromic approaches offered in IMCI, for example, is in the range and quality of the validation research undertaken prior to multicountry analysis [87-94] whereas only one published validation study of IMAI Acute Care has been done. More validation work is needed in a range of epidemiological settings, to improve the adult and adolescent integrated syndromic protocols for the care and treatment of common primary care conditions. Thus, IMAI and all integrated models should undergo rigorous validation against gold-standard assessment and treatment, thereby identifying the most sensitive and specific protocols, and highlighting opportunities for developing new approaches where gaps or weaknesses exist.

4. Multicountry evaluation: to be brought to scale and to have maximum impact on patient and population outcomes, integrated models of care must be implemented in a range of settings with varying epidemiology, health infrastructure and health workforce dynamics. Subsequently we must measure and define the impact of these models on the quality of care, health worker performance and process outcomes, and short-term outcomes for specific illnesses. More importantly, long-term effectiveness studies must be employed to define the impact of integrated clinical management on overall morbidity and mortality.

5. Cost and efficiencies: as with any successful program, we must examine the costs of implementing integrated models of primary care in poor and rural health systems in the developing world. As with many interventions based on education and feedback [8], we would anticipate that the cost effectiveness of such models to be high, but this requires specific study. Additionally, research must be performed to examine gains in efficiency in health system performance, such as in the identification and triage of severely ill patients to higher levels of care, screening for and integration with chronic disease programs, establishing a well-functioning referral network from health centers to hospitals at the district, and creating integrated electronic medical records and health information systems to support the delivery of high-quality primary care. As well, studies should be performed on the requisite staffing and human resources required to successfully implement integrated models of care such as IMAI.

6. Strengthening the integrated system: the success of integrated primary care delivery rests on its ability to function within a well-performing and integrated health system. Thus efforts to strengthen clinical care through integrated management must be complemented by a broader operational and policy strategy that addresses, for example, the use of information technology in the primary healthcare system to support delivery of care, the strengthening of management and administration capacity at facilities and district/regional public health authorities, the systematic integration of community health workers with health-center-based teams, and further upstream, the integration of financing of healthcare delivery at the front lines of the health system. Without these parallel interventions, any integrated clinical model cannot realize its full potential impact in improving primary care more broadly.

## Summary

In the developed world, integrated primary care is a given. Primary care providers are expected to be able to triage, screen for, diagnose, and manage a wide range of conditions, and when they cannot, to know when and to whom to refer for specialty consultation or more advanced or intensive care. This expectation is set by society, and reflected by the profession through the education and training of front-line providers. It is towards this model that we should aim globally, and perhaps most vitally in the poorest and most rural of settings where structural inequality and relative geographic isolation make high-quality, front-line primary care even more necessary.

We recognize that disease-specific programs are essential components of global primary care. And of course, comprehensive primary healthcare cannot be reduced to the ambulatory clinical services provided to a select population. But neither can efforts to strengthen the quality of primary care delivery in the developing world continue to rely on vertical programs alone to drive improvement. Instead, a paradigm shift is required that will give providers a sophisticated and standard way to approach each patient; one that offers robust and sensitive screening methodologies, that integrates care for acute and chronic conditions as well as prevention and treatment, and that strengthens referral and follow-up patterns as systems transition toward longitudinal care of the patient. Integrated models of primary care such as IMAI provide a clear approach that can be iterated and improved upon to that end. Going forward, successful integrated models of care must come from the people and places where they will be delivered, given the proximity of primary care delivery to the communities served. Thus with a commitment to innovating, implementing, and improving upon models of integrated care, including IMAI, we can finally deliver on the promise of Alma-Ata.

## Abbreviations

AIDS: Acquired immune deficiency syndrome; ART: Antiretroviral therapy; HIV: Human immunodeficiency virus; IMAI: Integrated Management of Adult & Adolescent Illness; IMCI: Integrated Management of Childhood Illness; MDGs: Millennium Development Goals; NCD: Non-communicable disease; OPD: Outpatient department; PALSA: Practical Approach to Lung Health in South Africa; STI: Sexually transmitted infection; TB: Tuberculosis; UNICEF: United Nations International Children’s Emergency Fund; WHO: World Health Organization.

## Competing interests

All authors declare that they have no competing interests, financial or otherwise, in relation to this research or production of this manuscript.

## Authors’ contributions

AV led the initial design and conception of the manuscript with support from SDL and DCM and led the writing of the first and all subsequent drafts of the manuscripts, including incorporation of coauthors’ edits. AE worked directly with AV on design and conception of the manuscript and contributed significant written and editorial inputs to the manuscript at every stage. SDL supported AV and AE in the design and conception of the manuscript and contributed substantive editorial inputs to early drafts of the manuscripts. NG and MA worked together and contributed significant research and substantive editorial inputs to early drafts of the manuscript, especially the Background. PD and TN contributed substantive editorial inputs to later drafts of the manuscript especially the Discussion. SG and KS contributed significant research and editorial input to the Discussion, especially the sections on IMAI, with SG also contributing to the sections on IMCI and NCDs. DCM and PEF served as the senior authors on the manuscript, providing overall editorial guidance to the lead authorship team and contributing significant editorial and intellectual content to later drafts of the manuscript. PEF in particular contributed significant edits to the final version. All listed authors gave final approval to the manuscript.

## Authors’ information

AV, SG, and KS are former staff members at the World Health Organization in Geneva, and former members of the IMAI Team within the Department of HIV/AIDS; SG was the former WHO IMAI team leader and currently Executive Director of the IMAI-IMCI Alliance AE is currently the Director of the Program in Primary Care and Social Change in the Department of Global Health and Social Medicine at Harvard Medical School, where AV and PEF are affiliated, and where PEF is the Department Chairman and Kolokotrones University Professor of Global Health and Social Medicine. SDL and DCM are faculty members in the Department of Clinical Research, Faculty of Infectious and Tropical Diseases at the London School of Hygiene & Tropical Medicine, where DCM is the Dean of the Faculty and AV is a PhD Candidate. MA, NG, and PD are currently part of the Partners In Health/*Inshuti Mu Buzima* team in Rwanda, where PD is the Country Director and where AV was previously employed and is currently affiliated. TN is a former employee at Partners In Health, Boston, and KS is currently the Clinical Director at Partners In Health - Lesotho. AE, NG, KS, and PEF are clinical faculty at the Division of Global Health Equity at the Brigham & Women’s Hospital in Boston, USA. AV is also a Senior Resident Physician and Assistant Chief Resident for Primary Care in the Department of Medicine at Weill Cornell Medical College and New York-Presbyterian Hospital.
